# Indications and Findings of Upper Gastrointestinal Endoscopy at a District Hospital in Ghana: A Retrospective Study

**DOI:** 10.1002/hsr2.70504

**Published:** 2025-02-23

**Authors:** Samuel Kyeremeh Adjei, Prosper Adjei

**Affiliations:** ^1^ Department of Internal Medicine Methodist Hospital Wenchi Ghana; ^2^ School of Public Health and Allied Sciences Catholic University of Ghana Fiapre‐Sunyani Ghana

**Keywords:** dyspepsia, Ghana, indications, upper gastrointestinal endoscopy, upper GI bleeding

## Abstract

**Background and Aims:**

Gastrointestinal diseases are a significant global health issue, with symptoms from these conditions negatively affecting quality of life and causing substantial economic consequences. Esophagogastroduodenoscopy, also referred to as upper gastrointestinal endoscopy, is an important and safe procedure used for both diagnosis and treatment in the field of gastroenterology. This study sought to assess the characteristic clinical indications and endoscopic findings of individuals presenting at Methodist Hospital, Wenchi, Ghana.

**Methods:**

A retrospective analysis was conducted by examining the medical records of patients who underwent upper gastrointestinal endoscopy from April 2023 to June 2024.

**Results:**

Out of 405 patients who underwent upper gastrointestinal endoscopy during the study period, 396 were included in the subsequent analysis. The average age of patients was 43.7 years with a standard deviation of 16.6 and ranging from 12 to 100 years. Approximately 42.9% of the patients were males. Dyspepsia was the most common reason for upper gastrointestinal endoscopy (60.9%), followed by retrosternal chest pain (17.7%), heartburn (12.1%) and upper gastrointestinal bleeding (3.8%). Results of upper gastrointestinal endoscopy were normal in 15.2% of patients. The most prevalent pathologies of the esophagus, stomach and duodenum were gastroesophageal reflux disease (17.4%), gastritis (31.8%), and duodenitis (4.5%), respectively.

**Conclusion:**

Dyspepsia was the primary indication for endoscopic evaluation of the upper gastrointestinal tract. The most prevalent pathological finding was gastritis, followed by gastroesophageal reflux disease, gastric ulcer, hiatal hernia and duodenitis. Esophagogastroduodenoscopy is an essential investigative tool for diagnosing upper gastrointestinal tract pathologies.

**Abbreviations**
GERD: gastroesophageal reflux disease
SD: standard deviation
UGI: upper gastrointestinal
UGIE: upper gastrointestinal endoscopy

## 1 Introduction

Among the most frequent complaints that drive patients to seek medical attention are upper gastrointestinal (UGI) symptoms, which are especially common in the general populace [1, 2]. Diseases related to the UGI tract significantly contribute to global morbidity and mortality, affecting millions of individuals worldwide [3]. Key procedures such as the upper gastrointestinal endoscopy (UGIE), allows for the visualization of the upper part of the gastrointestinal tract using a flexible endoscope passed through the mouth into the esophagus and extending to the second part of the duodenum. This procedure enables real‐time evaluation and interpretation of the observed findings. UGIE with biopsy is widely acknowledged as the gold standard for diagnosing UGI tract malignancies like gastric tumors and esophageal adenocarcinoma. Beyond facilitating accurate diagnosis of various UGI disorders, UGIE also presents opportunities for therapeutic interventions through minimally invasive techniques [4, 5, 6]. This procedure is frequently performed and provides valuable insights into patients with gastrointestinal issues, yielding superior diagnostic outcomes compared to radiological methods, particularly in cases of UGI bleeding and inflammatory conditions such as esophagitis, gastritis and duodenitis [7].

Key reasons for performing UGIE include assessing persistent upper abdominal symptoms despite appropriate treatment, upper abdominal symptoms accompanied by alarm features (such as weight loss, dysphagia, anemia, unexplained persistent vomiting, melena stools and hematemesis), incipient dyspepsia in individuals over 50 years of age and unremitting esophageal reflux. It is employed for the surveillance of malignancies including familial adenomatous polyposis coli syndrome, adenomatous gastric polyps, Barrett's esophagus and gastric or esophageal ulcers. Furthermore, UGIE is intended for the screening of esophageal varices in cirrhotic patients, tracing occult gastrointestinal bleeding, evaluating caustic substance ingestion and assessing chronic diarrhea [8, 9].

Although there is a wealth of literature on indications and findings of UGIE in teaching and some regional hospitals across the country [10, 11, 12, 13] there is paucity of literature on UGIE from district hospitals, which mostly offer primary healthcare services. This study sought to explore the clinical indications and endoscopic findings of patients who underwent UGIE at a district hospital in Ghana.

## 2 Materials and Methods

### 2.1 Study Design and Setting

This study conducted a retrospective analysis of patients who underwent UGIE at Methodist Hospital, Wenchi, Ghana, between April 2023 and June 2024. The endoscopy unit at the hospital commenced operations in April 2023 and follows an open access policy, allowing patients to be referred directly to the unit from various hospitals located within Wenchi Municipality and its surrounding areas.

### 2.2 Study Population

All patients who underwent UGIE for various indications during the study period were included in the study, with the exception of those whose data were significantly incomplete, inconsistent or illegible.

### 2.3 Equipment and Procedure

The UGIE procedure was conducted following established protocols using Sonoscape HD 500 Endoscope‐EG‐500L (Sonoscape Medical Corporation, Resolution‐1080 pixel, Full HD:60FPS, HDL‐500X Xenon light source, VIST Chromoendoscopy, Water Jet). Before the intervention, patients were required to fast for at least 6 h and a comprehensive preprocedural evaluation was carried out for each patient. To enhance patient comfort, a 10% lignocaine spray was applied to anesthetize the pharynx in all instances. Furthermore, sedation was achieved through the administration of 2.5 mg of intravenous midazolam. To promote smooth muscle relaxation and mitigate gastrointestinal spasms, intravenous hyoscine butylbromide (Buscopan) was also administered. Although the rapid urease test for Campylobacter‐like organisms was not conducted, *Helicobacter pylori* infections were identified using the *H. pylori* stool antigen test, which demonstrated a specificity of 93.8% and a sensitivity of 96.7% (CTK Biotech Inc., USA). Children younger than 12 years and unstable patients were excluded from the examination. Mucosal biopsies for histological analysis were taken where necessary. The procedures were performed by a specialist general surgeon and an internal medicine specialist with training and experience in UGIE.

### 2.4 Data Retrieval, Processing and Analysis

Data was obtained from endoscopy registers and patient medical records. The information was entered into Microsoft Excel and subsequently analyzed using Statistical Package for Social Sciences (SPSS) version 25. The records included details on patient age and gender, the primary reason for the procedure and key endoscopic findings. Ethical approval as well as waiver of informed consent was obtained from the Research Committee (Ethics approval reference: MHW/RP/103). Descriptive statistics were reported as frequencies and percentages.

## 3 Results

During the study period, a total of 405 patients underwent esophagogastroduodenoscopy for various clinical indications. Of these, 396 patients were included in the analysis, while nine were excluded due to incomplete data. Among the included patients, 42.9% (*n* = 170) were males and 57.1% (*n* = 226) were females. The average age of the patients was 43.7 years (SD ± 16.6) and ranged from 12 to 100 years. Notably, 52% (*n* = 206) of the patients were over 40 years old. The highest frequency of endoscopic procedures was observed in the 31–45 age group (Table 1).

**Table 1:** Age and gender distribution of patients who underwent upper gastrointestinal endoscopy at Methodist Hospital, Wenchi, Ghana, (*n* = 396).

Characteristic	Frequency (*n*)	Percentage (%)
Age (years)		
Mean ± SD	43.7 ± 16.6	
Range	12–100	
Age group		
12–20	24	6.1
21–30	70	17.7
31–40	96	24.2
41–50	84	21.2
51–60	51	12.9
61–70	43	10.9
71–80	20	5.1
> 80	8	2.0
Gender		
Male	170	42.9
Female	226	57.1

Abbreviation: SD, standard deviation.

Dyspepsia, characterized by upper abdominal pain or discomfort [14] was the predominant reason for undergoing UGIE, accounting for 60.9% (*n* = 241) of cases. This was followed by retrosternal chest pain at 17.7% (*n* = 70), heartburns at 12.1% (*n* = 48), and UGI bleeding at 3.8% (*n* = 15). Less frequent indications included chemical ingestion (0.3%), suspicion of malignancy (0.3%), and foreign body ingestion (0.5%) (Table 2).

**Table 2:** Indications for upper gastrointestinal endoscopy at Methodist Hospital, Wenchi, Ghana, (*n* = 396).

Indication	Frequency (*n*)	Percentage (%)
Chemical ingestion	1	0.3
Dysphagia	8	2.0
Dyspepsia	241	60.9
Heartburns	48	12.1
UGI bleeding	15	3.8
Ingestion of foreign body	2	0.5
Retrosternal chest pain	70	17.7
Suspicion of malignancy	1	0.3
Persistent vomiting	10	2.5

Abbreviation: UGI, upper gastrointestinal.

Among patients presenting with dyspepsia, the most frequently identified pathologies were gastritis 46.5% (*n* = 112), gastroesophageal reflux disease (GERD) 12.4% (*n* = 30), and gastric ulcer 10% (*n* = 24). Normal endoscopic findings were observed in 15.8% (*n* = 38) of dyspeptic patients.

Regarding patients with UGI bleeding, 66.7% (*n* = 10) were found to have esophageal varices, followed by gastric ulcer 20% (*n* = 3), duodenal ulcer 6.7% (*n* = 1) and gastritis 6.7% (*n* = 1).

Normal findings were reported in 15.2% (*n* = 60) of the UGI endoscopic examinations, while pathologies were detected in 84.8% (*n* = 336), with a majority (68.3%) being male patients (Table 3).

**Table 3:** Endoscopic findings of patients who underwent upper gastrointestinal endoscopy at Methodist Hospital, Wenchi, Ghana, (*n* = 396).

Endoscopic finding	Overall (*n* = 396)	Male (*n* = 170)	Female (*n* = 226)	Age (< 40) (*n* = 190)	Age (> 40) (*n* = 206)
Esophagus					
Esophageal candidiasis	5 (1.3)	3 (60.0)	2 (40.0)	2 (40.0)	3 (60.0)
Esophagitis	11 (2.8)	5 (45.5)	6 (54.5)	6 (54.5)	5 (45.5)
Sliding hiatal hernia	27 (6.8)	13 (48.1)	14 (59.1)	11 (40.7)	16 (59.3)
GERD	69 (17.4)	26 (37.7)	43 (62.3)	33 (47.8)	36 (52.2)
Esophageal varices	16 (4.0)	14 (87.5)	2 (12.5)	6 (37.5)	10 (62.5)
Esophageal ulcer	2 (0.5)	1 (50.0)	1 (50.0)	0 (0.0)	2 (100)
Esophageal polyp	2 (0.5)	1 (50.0)	1 (50.0)	0 (0.0)	2 (100)
Stomach					
Gastritis	126 (31.8)	46 (27.1)	80 (72.9)	73 (57.9)	53 (42.1)
Gastric ulcer	34 (8.6)	22 (64.7)	12 (35.3)	8 (23.5)	26 (76.5)
Gastric polyp	5 (1.3)	2 (40.0)	3 (60.0)	0 (0.0)	5 (100)
Gastric cancer	5 (1.3)	4 (80.0)	1 (20.0)	1 (20.0)	4 (80.0)
Duodenum					
Duodenitis	18 (4.5)	6 (33.3)	12 (66.7)	8 (44.4)	10 (55.6)
Duodenal ulcer	16 (4.0)	8 (50.0)	8 (50.0)	6 (37.5)	10 (62.5)
Normal findings	60 (15.2)	19 (31.7)	41 (68.3)	36 (60.0)	24 (40.0)

Abbreviation: GERD, gastroesophageal reflux disease.

Histopathology results were not available during the study period. However, *H. pylori* stool antigen test results were available for 120 patients, out of which 98 were positive. The prevalence of *H. pylori* infection was 81.7%. Among patients with *H. pylori* infection, 69.4% (*n* = 68) had gastritis, followed by gastric ulcer 18.4% (*n* = 18), duodenal ulcer 7.1% (*n* = 7), and duodenitis 5.1% (*n* = 5).

### 3.1 Esophagus

Esophageal candidiasis, described as infection within the esophagus caused by *Candida albicans* and resulting in the macroscopic appearance of mucosa covered with white plaque‐like lesions that overlie a friable and erythematous mucosa [15] was found in 1.3% of patients with a higher incidence in males. Esophagitis, defined as the inflammation of the esophageal lining [16] was identified in 2.8% of patients, with females making up the majority at 54.5%. The occurrence of sliding hiatal hernia, defined as a separation greater than 2 cm between the caudally displaced esophagogastric junction and the diaphragmatic impression [17], was observed in 6.8% (*n* = 28) of patients, with a greater prevalence in females. Approximately 17.4% of patients were diagnosed with GERD, indicated by inflammation (erythema) in the lower esophagus and/or a relaxed lower esophageal sphincter [18]. Esophageal varices were found in 4.0% of patients, predominantly affecting males (8.5%). Less common pathologies of the esophagus found were esophageal ulcer and esophageal polyp (Table 3).

### 3.2 Stomach

Gastritis, characterized by the inflammation of the gastric mucosa and identified endoscopically by signs such as reddening or edema of the mucosal lining [19], was observed in 31.8% (*n* = 126) of the patient population. In contrast, gastric ulcers were present in 8.6% (*n* = 34) of patients, predominantly affecting males. Instances of gastric cancer and gastric polyps were noted in a limited number of cases.

### 3.3 Duodenum

Duodenitis was identified in 4.5% (*n* = 18) of the patient population with a higher prevalence observed among females. Conversely, the occurrence of duodenal ulcers was evenly distributed among male and female patients.

## 4 Discussion

Esophagogastroduodenoscopy is widely regarded as the definitive investigative modality for conditions affecting the UGI tract [20]. During the study period, a total of 405 patients underwent this procedure, a noteworthy figure for a facility in its inaugural year of operation. The average age of patients who received esophagogastroduodenoscopy in this study was 43.7 years, which aligns with findings from other studies indicating that the mean age of individuals undergoing this procedure for diverse indications falls within the fourth decade of life [21, 22]. In the region where this study was conducted, farming is the predominant economic activity with the mean age of farmers in this area reported to be 42.6 years [23]. Due to work‐related musculoskeletal disorders, the abuse of over‐the‐counter nonsteroidal anti‐inflammatory drugs is quite common among farmers in Ghana, and this predisposes them to developing gastrointestinal symptoms which may require further assessment with UGIE. This probably is the reason for the average age (i.e., 43.7 years) of the participants in the current study. The participants in this study were aged between 12 and 100 years, which is comparable to the age range of 8–100 years reported in a Nigerian study [24]. While this study observed a female preponderance, other studies have indicated a predominance of male patients [25, 26]. Compared to their male counterparts, females in Ghana generally are more health conscious and open to discussing health‐related issues with healthcare professionals. Again, official data suggests that females account for 58.6% of the active membership of the National Health Insurance Scheme (NHIS) [27]. For these reasons, Ghanaian females experiencing gastrointestinal symptoms are more likely to seek medical care and subsequently undergo endoscopic evaluation if indicated.

Dyspepsia emerged as the predominant reason for UGIE among the majority of our patients. This finding aligns with studies conducted in Ghana and various other African nations, which similarly identified dyspepsia as the leading indication for endoscopic procedures [12, 28, 29, 30, 31]. Dyspepsia is a nonspecific gastrointestinal complaint which is frequently encountered in medical practice. Although it may be bothersome, it is often associated with normal endoscopic findings and continues to be a key indication for UGIE to rule out more sinister conditions like malignancies.

In this study, additional indications for UGIE included retrosternal chest pain, heartburns, UGI bleeding and persistent vomiting. Notably, dysphagia accounted for 2.0% of the patients who underwent UGIE, a figure that closely aligns with the 2.28% reported in a study from Ghana [13]. This differs from the findings of a study conducted in Southern Africa, which revealed that 37% of patients underwent UGIE as a result of dysphagia [32]. Variations in study populations, referral patterns and clinical guidelines may account for the discrepancies.

The current investigation identified gastritis as the predominant pathological finding, occurring in 31.8% of gastric endoscopies. This observation is consistent with studies conducted in Uganda and Saudi Arabia [33, 34], which also reported gastritis as the most frequently observed endoscopic finding. Frequent use of nonsteroidal anti‐inflammatory drugs, excessive alcohol consumption, smoking and prevalence of *H. pylori* infection in both developing and developed countries may account for this similarity. Gastric ulcer, representing the second most prevalent pathological condition of the stomach at a rate of 8.6%, was identified more often than duodenal ulcers, which occurred at a rate of 4.0% within our patient population. This finding aligns with a study conducted in Kumasi, Ghana [12]. This finding, in contrast, diverges from the outcomes of a comparable investigation carried out in Accra, Ghana, where a higher incidence of duodenal ulcers was reported [35]. This disparity may be ascribed to the fact that the current study was conducted in a district where the residents are predominantly farmers and are more likely to abuse nonsteroidal anti‐inflammatory drugs after working on their farms. The most common pathological finding of the duodenum was duodenitis, occurring in 4.5% of cases, a rate that is lower than that reported in comparable studies [36, 37]. Differences in study population characteristics and variations in geographical settings may account for the disparities.

Regarding the esophagus, GERD emerged as the most significant pathological finding, succeeded by sliding hiatal hernia. The incidence of hiatal hernia observed in this study was 6.8%, aligning closely with results documented in a Nigerian study [31]. Shared dietary habits such as low‐fiber diets and common lifestyle factors such as obesity and sedentary behaviours among these West African countries may contribute to this similarity.

In relation to gastrointestinal malignancies, the current study observed a gastric cancer prevalence of 1.3%, which is marginally lower than the 2.1% prevalence reported in a study conducted in Ghana [12]. Regional variations in lifestyle and dietary patterns as well as differences in population characteristics are likely to influence this outcome. Additionally, the incidence of normal endoscopic findings was notably lower compared to earlier studies conducted in Ghana [12, 35]. Advances in endoscopic equipment, techniques and changes in diagnostic criteria over time may affect the detection of subtle lesions or conditions that may have previously been classified as normal.

The findings of this study indicate that UGI pathologies were primarily observed in individuals over the age of 40, representing 54.2% of the pathological results obtained. This observation aligns with a comparable study conducted in a different setting, which noted that 59.7% of pathological findings were present in patients aged over 40 [38]. The observed similarity in findings can be attributed to demographic resemblance, shared risk factors including dietary practices and lifestyle choices, as well as comparable healthcare systems. *H. pylori* inhabitation of the gastrointestinal tract is one of the most prevalent infections worldwide, serving as the primary cause of chronic gastritis and a significant contributor to gastric cancer and peptic ulcer disease [39, 40]. This study found the prevalence of *H. pylori* infection to be 81.7%, surpassing the rates observed in previous studies conducted in the country [41, 42]. The possible reasons for the difference may be the low socioeconomic status as well as poor sanitation and hygiene practices among inhabitants of the study area.

The notably high prevalence of esophageal varices observed in this study reflects a significant burden of chronic liver disease secondary to chronic hepatitis B viral infection in the Bono Region and Ghana at large. The prevalence of hepatitis B infection in the Bono region is estimated to be between 10.2% and 14.6% [43, 44]. At the national level, the prevalence rate of hepatitis B infection is estimated at 12.3% to 14.4%, placing Ghana as a highly endemic country [45, 46]. Prevention of mother to child transmission, intensification of the expanded program on immunization, screening and vaccination of adults at risk of hepatitis B viral infection, as well as early diagnosis and appropriate treatment are some of the recommended strategies aimed at reducing the prevalence of chronic hepatitis B viral infection and its attendant complications.

The high prevalence of gastritis and GERD observed in this study carries significant clinical and public health implications, particularly in resource‐limited settings. These findings underscore the need for targeted strategies to address both the diagnosis and management of these conditions, as well as the implementation of effective preventive measures. Training of endoscopists and investments in endoscopy facilities at the district level by hospital managers and government are required to enhance early detection and treatment of these conditions. Sensitization campaigns aimed at addressing risk factors such as excessive alcohol consumption, smoking and abuse of nonsteroidal anti‐inflammatory drugs could play a pivotal role in reducing the incidence of gastritis and GERD. Furthermore, improving sanitation and access to clean water may help reduce the transmission of *H. pylori*, particularly in populations where it remains highly prevalent.

The main limitation of this study was the unavailability of histopathology results during the analysis. Although suspicious lesions were biopsied during endoscopy, the patients had to take the specimens to other laboratories as our hospital laboratory is currently not equipped to perform histopathological examinations. This limitation may have resulted in overdiagnosis or underdiagnosis of certain gastrointestinal disorders. Without histopathological examination, gastrointestinal malignancies with subtle or atypical features may be misdiagnosed as benign conditions. Again, the reliance on endoscopic findings alone may contribute to the overdiagnosis of gastritis and reflux esophagitis, as the diagnosis of these conditions is based on visual inspection and can potentially be influenced by the subjective interpretation of endoscopists. The reliance on visual assessment alone can lead to diagnostic inaccuracies. Therefore, the findings of this study should be viewed within this context. Despite this limitation, the study provides meaningful perspectives of the burden and patterns of UGI diseases in a resource‐limited setting.

## 5 Conclusion

5.1

This study emphasizes the vital role of UGIE in a district hospital setting, highlighting its indispensable value in diagnosing and managing UGI disorders. Dyspepsia emerged as the predominant reason for UGIE in this study, with gastritis identified as the most prevalent endoscopic finding. Additionally, gastric ulcers were observed more frequently than duodenal ulcers among the patient population. The prevalence of *H. pylori* infection was significantly higher compared to other studies, underscoring the need for screening for *H. pylori* in patients with dyspeptic symptoms and the initiation of effective eradication therapy to reduce infection rates. Again, this study not only highlights the common indications and significant findings associated with the procedure, but also reinforces the need for its continued integration into district hospital services to optimize patient care and resource utilization.

## Author Contributions

**Samuel Kyeremeh Adjei:** conceptualization, formal analysis, writing–original draft, writing–review and editing, data curation. **Prosper Adjei:** writing–review and editing.

## Ethics Statement

Ethical approval was obtained from the Research Committee of Methodist Hospital Wenchi before commencement of the study (Ethics approval reference: MHW/RP/103). The study did not incorporate any identifying details such as patient name or identification number, ensuring that all data was handled with the highest level of confidentiality.

## Consent

Additionally, the requirement for informed consent was waived by the Research Committee.

## Conflicts of Interest

The authors declare no conflicts of interest.

## Transparency Statement

The lead author Samuel Kyeremeh Adjei affirms that this manuscript is an honest, accurate, and transparent account of the study being reported; that no important aspects of the study have been omitted; and that any discrepancies from the study as planned (and, if relevant, registered) have been explained.

## Data Availability

The datasets used and/or analyzed during this study are available from the corresponding author upon reasonable request.
